# An Approach toward the Realization of a Through-Thickness Glass Fiber/Epoxy Thermoelectric Generator

**DOI:** 10.3390/ma14092173

**Published:** 2021-04-23

**Authors:** George Karalis, Christos K. Mytafides, Lazaros Tzounis, Alkiviadis S. Paipetis, Nektaria-Marianthi Barkoula

**Affiliations:** Department of Materials Science and Engineering, University of Ioannina, 45110 Ioannina, Greece; gkaralis@uoi.gr (G.K.); cmytafides@uoi.gr (C.K.M.); latzounis@uoi.gr (L.T.); paipetis@uoi.gr (A.S.P.)

**Keywords:** glass fiber-reinforced polymer composite, multifunctional structural laminate, thermal energy harvesting, through-thickness thermal gradient, thermoelectric generator (TEG)

## Abstract

The present study demonstrates, for the first time, the ability of a 10-ply glass fiber-reinforced polymer composite laminate to operate as a structural through-thickness thermoelectric generator. For this purpose, inorganic tellurium nanowires were mixed with single-wall carbon nanotubes in a wet chemical approach, capable of resulting in a flexible p-type thermoelectric material with a power factor value of 58.88 μW/m·K^2^. This material was used to prepare an aqueous thermoelectric ink, which was then deposited onto a glass fiber substrate via a simple dip-coating process. The coated glass fiber ply was laminated as top lamina with uncoated glass fiber plies underneath to manufacture a thermoelectric composite capable of generating 54.22 nW power output at a through-thickness temperature difference οf 100 K. The mechanical properties of the proposed through-thickness thermoelectric laminate were tested and compared with those of the plain laminates. A minor reduction of approximately 11.5% was displayed in both the flexural modulus and strength after the integration of the thermoelectric ply. Spectroscopic and morphological analyses were also employed to characterize the obtained thermoelectric nanomaterials and the respective coated glass fiber ply.

## 1. Introduction

Nowadays, there is a continuously increasing rate of global energy consumption. Although efforts have been made toward the exploitation of renewable or alternative energy sources, their use is still limited [1]. Moreover, eco-friendly solutions are required not only in terms of the source of energy but also in the way the power is supplied. For example, low power-consuming electronics such as wireless sensor networks typically use batteries as a power source. The limited lifetime of batteries results in increased total costs associated with their replacement in remote areas [2,3]. Next to that, high amounts of energy losses, often in the form of heat, could be partially recovered as power by proper energy conversion methodologies [4]. A promising way toward eco-friendly and autonomous structures is, thus, to broadly embed self-powered energy harvesting solutions, such as solar cells, vibration-based or thermal energy harvesters in structural materials [5,6,7,8,9]. This could be extremely relevant for boilers and steam piping systems, especially in large industrial and power plants, which show high amounts of wasted heat [10]. As a consequence, a reduction of operating and control costs could be achieved, also contributing to the new global requirements for CO_2_ emissions reduction [11].

The basic principle behind thermal energy harvesting is the thermoelectric effect (i.e., the Seebeck effect). It is well known that when a thermoelectric (TE) material is exposed to a temperature difference (Δ*T*), it spontaneously generates a potential difference (Δ*V*) due to the motion of free electrons (n-type semi-conductor material) or holes (p-type) toward specific directions. The magnitude of the Seebeck effect is expressed by the Seebeck coefficient (*S*), which is employed for the estimation of the power factor (*PF*) (see Equations (1) and (2)). This quantity is used for the direct comparison of various materials’ TE efficiency. The overall TE performance is classified by the dimensionless figure of merit (*ZT*) (see Equation (3)).

(1) $$S=-\frac{\Delta V}{\Delta T}$$

where:

*S* = the Seebeck coefficient in μV/K
Δ*V* = the generated TE voltage in mV
Δ*T* = the externally applied temperature difference in K

(2) $$PF=\sigma \times S^{2}$$

where:

*PF* = the power factor in μW/mK^2^
*σ* = the electrical conductivity in S/m

(3) $$ZT=\frac{\sigma \times S^{2}\times T}{\kappa }$$

where:

*ZT* = dimensionless figure of merit
*T* = the absolute temperature
*κ* = thermal conductivity in W/m·K

Depending on their intrinsic carrier mobility and concentration, efficient TE materials present high values of *ZT*, combining high electrical and low thermal conductivity (*κ*) [12,13]. Traditional TE materials typically consist of low bandgap semiconductors, e.g., Te, Bi_2_Te_3_, PbTe, etc. [14,15]. Recently, hybrid or organic nanostructured materials have been suggested as auspicious candidates for TE applications [16,17]. The scientific community is highly interested in blends of conductive polymers with inorganic thermoelectric crystals, bulk and 1-D superlattice nanostructures, etc., due to their ability to tune the carrier transport via, i.e., energy filtering mechanisms, inherent low thermal conductivity, tailored electrical conductivity, facile processing, relatively moderate large-scale production cost and superior flexibility properties [18,19,20].

Large-scale TE energy harvesting and conversion to sustainable electrical power is realized by TE generators (TEGs) devices. A common TEG device comprises single-type, or p-/n-type thermoelements interconnected electrically in series and thermally in parallel. Advancements in flexible and wearables TEGs have been recently reported, presenting desired power output values for practical applications [21,22,23,24,25]. Scientific works related to bulk or structural TEGs targeting different application areas have also been published [26,27,28]. Special interest has been concentrated on polymer nanocomposites [29,30,31], and fiber-reinforced polymer (FRP) composites [32,33] since such materials are widely used in aerospace, automotive, renewable energy, etc. applications. FRPs offer the potential for flexible design and novel manufacturing approaches with significantly improved specific properties, such as high strength to weight ratio [34,35]. Additionally, a variety of secondary functionalities can be introduced, transforming these materials into smart and multifunctional structures. This can be realized via the integration of dispersed nanomaterials in a polymer matrix or through hierarchical coatings deposited onto the reinforcing phases, such as glass and carbon fibers. Functionalities may include increased interfacial adhesion strength [36], increased interlaminar shear strength [37,38], non-destructive structural diagnostics [39,40], self-healing perspectives [41], energy storage capabilities [42], lightning-strike protection [43] and energy harvesting solutions [28].

More specifically, in the area of structural polymer composites, research has focused on the targeted enhancement of FRP’s TE properties through the introduction of nanomaterials [44]. Previous studies on in-plane and through-thickness TE properties of FRP laminates mainly focused on the polymer matrix-interfaces modification with nano or micro-scale fillers [45]. For instance, Han et al. reported increased Seebeck coefficient values from 8 to 163 μV/K by brushing a mixture of tellurium and bismuth microparticles onto carbon fiber prepregs of a polymer matrix-based structural composite [46].

Based on the above, the existing literature findings are limited to the bulk through-thickness interface modifications, mainly with inorganic microparticles, to achieve enhanced TE response at the laminate level. Instead of modifying the matrix and/or the interface of a structural composite, the main goal of the current work is to develop, for the first time, an approach that involves the integration of a proper architecture acting as a through-thickness structural TEG device in a composite laminate. To achieve this goal, an inorganic-organic nanomaterial based on tellurium nanowires (TeNWs) with single-wall carbon nanotubes (SWCNTs) added during growth is deployed. The nanostructured TE material is produced following a surfactant-assisted chemical reduction reaction based on previously well-established synthetic routes [47,48,49] with a few variations. The rationale behind this selection is to obtain enhanced performance through the combination of the TE properties of the two nanomaterials and flexibility at a film level via the use of highly durable SWCNTs. By redispersing the synthesized nanomaterials, an aqueous TE ink is prepared and used to coat a glass fiber (GF) unidirectional (UD) fabric via a simple dip-coating and oven-drying process. Eventually, the coated ply is purposely laminated to manufacture a 10-ply GF reinforced polymer (GFRP) laminate. The structure of the obtained nanomaterials is characterized using Raman spectroscopy and X-ray diffraction (XRD) analyses. Successful GF coating is confirmed via scanning electron microscopy (SEM). The TE performance of the nanomaterials and TEG laminates is also assessed. Finally, the effect of the TE ply integration on the mechanical performance of the obtained laminates is investigated under flexural loading. The obtained results reveal that it is possible to modify a conventional thin laminate with inorganic-organic nanomaterials on a ply level to enable efficient through-thickness thermal energy harvesting capabilities without eliminating the structural integrity of the obtained structure. Thus, the current paper deals with the demonstration of the ability of FRPs to act, by design, as through-thickness TEGs, with the aim to harvest thermal energy during the operational lifetime in the presence of temperature gradients.

## 2. Materials and Methods

### 2.1. Materials

For the preparation of the TE nanomaterials, ascorbic acid (AA) with 99% purity, sodium dodecylbenzenesulfonate (SDBS, 348.48 g/mol), and sodium tellurite (Na_2_TeO_3_) ~100 mesh with >99% purity were purchased from Sigma Aldrich (Missouri, USA). SWCNTs dispersion (TUBALL, INK H_2_O 0.2%) was acquired by OCSiAl (Novosibirsk, Russia). All chemicals were analytical grade and used as received without any further purification procedure. Distilled (DI) water was used throughout this research. PVDF membrane (pore size 45 μm) was purchased from Merck (Darmstadt, Germany) and used for the preparation of Te-based buckypapers.

For the manufacturing of the GFRP laminates, unidirectional (UD) glass fabric 320 gr/m^2^ with a single-ply thickness of 0.26 mm from Fibermax (Volos, Greece) was used. The epoxy resin system Araldite LY 5052/Aradur 5052 was purchased from Huntsman (The Woodlands, TX, USA) and was used as the matrix of the composite. To facilitate the fabrication of the TEG device, silver paste (ORGACON^TM^ Nanosilver Screen Printing Ink SI-P2000) was received from Agfa (Mortsel, Belgium), while silver foil tape (thickness of 0.055 mm) with conductive adhesive was acquired by 3M™ (Saint Paul, MN, USA).

### 2.2. Synthesis of TE Nanomaterial and Ink Preparation

**Figure 1:**
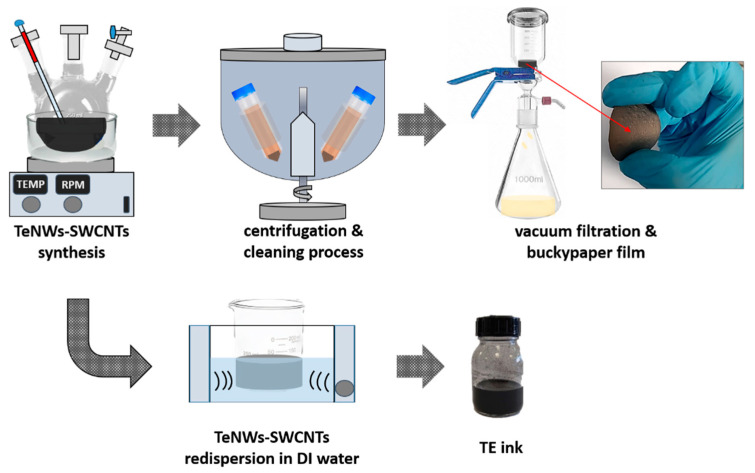
The steps followed for the synthesis of the inorganic-organic TE material and respective ink including: the solvothermal reaction step, the centrifugation and cleaning procedure with DI water, the vacuum filtration and buckypaper preparation procedure, the re-dispersion process, and the final TE ink.

The whole process, from the synthesis of the TE material to the formation of the TE ink is illustrated in Figure 1. Initially, TeNWs were synthesized according to the following procedure: 4.93 g AA was dissolved in 200 mL of DI water in a reaction flask followed by the addition of 0.10 g SDBS. SDBS has been introduced due to its high dispersion efficiency that results in the prevention of agglomeration phenomena [50,51]. After the homogenization of the solution, 0.28 g Na_2_TeO_3_ was added to the vigorously stirred mixture. For the synthesis of the inorganic-organic TE nanomaterial, 2.5 mL of the SWCNTs commercial ink was added to the previous mixture. Consequently, the mixture was raised up to 90 °C for 20 h and then left to cool down. The cleaning procedure included centrifugation at 8000 rpm for 30 min and removal of the sediment by dilution with DI water and pouring off repeatedly the SDBS rich supernatant side products and the residual reagents. The final precipitated material was redispersed and via a vacuum filtration process through a PVDF membrane filter (0.45 µm filter pore size) collected in the form of buckypaper while being kept finally for drying at 80 °C overnight. Finally, the buckypaper was redispersed in DI water (40 mg/mL) via bath-sonication for 30 min, resulting in a homogenous dispersion, hereafter denoted as TE ink. For comparison reasons, a buckypaper film was developed based on TeNWs before the in-situ growth in the presence of SWCNTs and used as reference material.

### 2.3. Manufacturing of the GFRP Laminate with the Through-Thickness TEG Functionality

**Figure 2:**
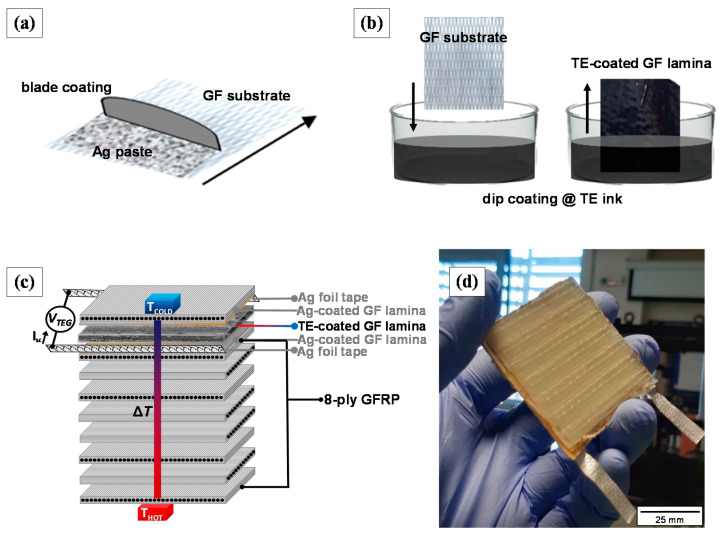
Schematic illustration of (**a**) blade coating for silver paste deposition onto the GF ply for the production of highly conductive electrode-like plies, (**b**) dip-coating of the GF ply within the TE ink to produce the coated functional ply, (**c**) the detailed lamination of the multifunctional GFRP and (**d**) photo of the manufactured through-thickness TEG GFRP laminate.

The fabrication of a single thermoelement TEG required firstly the integration of highly conductive electrode-like plies that will function as the interconnection between the internal TEG structure and the external electrodes. To do so, 2 GF plies were one-sided blade-coated with silver (Ag) paste, as shown schematically in Figure 2a. Then, the Ag-coated GF laminae were transferred in a ventilated oven and cured for 10 min at 150 °C. Afterward, Ag tape stripe was adapted to each Ag-coated GF lamina using a conductive adhesive to create the external electrodes. The next step was the incorporation of the TE functional ply into the laminate, as depicted in Figure 2b. This was achieved employing a facile dip-coating process, where the GF ply was immersed into the TE ink and subsequently dried overnight at 80 °C. For the manufacturing of the 10-ply GFRP TEG (50 × 50 mm^2^), the TE-coated GF ply was sandwiched between the Ag-coated GF plies to create the top layer of the composite, while 7 unmodified GF plies were added below the internal Ag-coated GF ply, following a cross-ply lamination (see Figure 2c). A plain 10-ply GFRP laminate was also developed for comparison. Both the TEG GFRP laminate, as well as the reference plain GFRP laminate, were manufactured by hand lay-up epoxy resin impregnation and thermopressing. According to the specifications of the thermoset system, the resin to hardener weight ratio was set at 100:38 w/w. Curing was conducted for 24 h at room temperature (R*T*) under 3 MPa pressure using a hydraulic press, and the post-curing was performed at 100 °C for 4 h. Based on the technical datasheet of the manufacturer, the T_g_ of the resin system, after this curing cycle, is in the range of 120 to 134 °C, while dynamic mechanical analysis unpublished data on the reference GFRP laminate (before the incorporation of the electrodes and the functional ply) indicated a T_g_ of 131 °C. Attention was paid to avoid any direct contact between the 2 Ag-coated GF laminae. This was ensured through the strict alignment of the functional GF ply during the fabrication of the TEG device. The manufactured GFRP TEG is presented in Figure 2d.

### 2.4. Characterization Methodologies

The structure of the synthesized inorganic-organic TE nanomaterial was characterized via Raman spectroscopy and XRD. Both Raman and XRD spectra were obtained from the TE buckypaper films. The spectroscopic measurements were carried out with a Labram HR (Horiba, Kyoto, Japan) scientific micro-Raman system. The 514.5 nm line of an Ar^+^ ion laser operating at a power of 1.5 mW at the focal plane was employed for the Raman excitation. An optical microscope equipped with a 50× long working distance objective served both for delivering the excitation light and collecting the back-scattered Raman light. Raman spectra in the range of 90–3500 cm^−1^ were collected. XRD analysis was performed with a D8 ADVANCE system (Bruker, Billerica, MA, USA) in symmetric step-scan mode with 2θ = 0.05° in transmission mode. The diffractometer operated at 40 kV and 30 mA with Kα radiation (λ = 1.5406 Å), diffraction angle (θ, 10° < 2θ < 80°), and a step size of 5° at room temperature. The morphology investigation of the coated GF ply was performed using JEOL JSM 6510 LV SEM/Oxford Instruments (JEOL, Tokyo, Japan) with an operating voltage of 3.5 kV.

**Figure 3:**
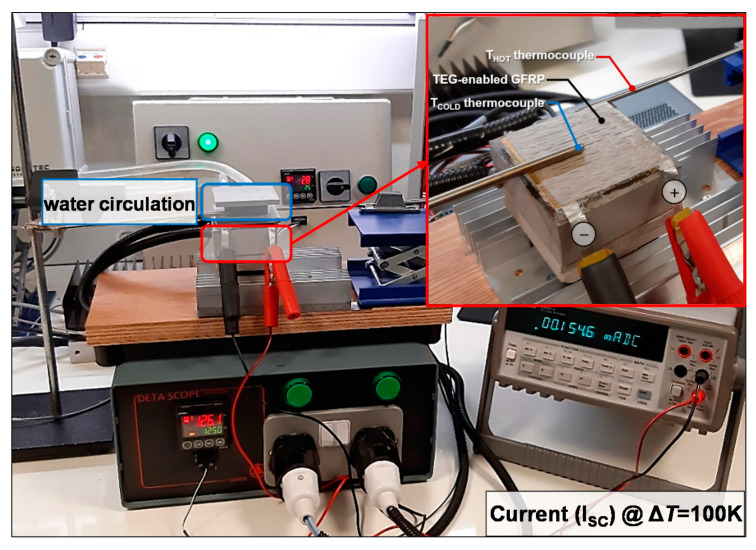
Demonstration of the through-thickness TEG GFRP TE performance during exposure to ∆*T* = 100 K.

The electrical resistivity values of the produced buckypaper films were obtained using a typical 4-probe sheet resistance commercial system (Ossila Ltd., Sheffield, UK). The generated TE voltage (Δ*V*) of the produced buckypaper films (in-plane) and the TEG laminate (through-thickness) was measured with a 34401A multimeter (Agilent, Santa Clara, CA, USA). As illustrated in Figure 2c, the voltage was measured using the metallic connectors-electrodes (Ag-coated GF laminae) located in the 8th and 10th plies of the TEG laminate. Thus, the through-thickness TE voltage output measurements were defined in the transverse direction of the device based on a ~0.27 mm interelectrode distance (thickness of the TE ply). A custom-made setup consisting of two metal blocks was developed for the generation of a temperature gradient (see Figure 3). For all measurements, one block was kept at room temperature (~25 °C) via water circulation, while the other was heated at higher temperatures via calibrated temperature-controlled resistors, allowing the generation of a Δ*T*. Three different levels of the thermal gradient were applied (i.e., Δ*T* of 50, 75, and 100 K), which result in temperatures below or close to the T_g_ of the TEG laminates to avoid any substantial degradation in the structural integrity upon heating. The temperature of the two blocks was constantly measured with K-type thermocouples. Figure 3 demonstrates the generated short-circuit current at Δ*T* of 100 K. Note that optical inspection for the TEG GFRP laminates indicated the absence of obvious evidence for any kind of degradation after several testing hours of continuous operation at the enforced maximum temperature gradient of 100 K.

This enables the calculation of *S*, *PF,* and *ZT* according to Equations (1)–(3).

Consequently, the thermal to electrical energy conversion efficiency (Carnot efficiency—*η*) can be determined by Equation (4) [52]:

(4) $$\eta =\left(\frac{T_{H}-T_{C}}{T_{H}}\right)\frac{\sqrt{1+\bar{ΖT}}-1}{\sqrt{1+\bar{ΖT}}+\left(T_{C}/T_{H}\right)}$$

where:

*T_H_* = the temperature of the hot side in K
*T_C_* = the temperature of the cold side in K
$\bar{ZT}\;$ = *ZT* calculated at the average temperature between the hot and the cold side

Based on the above measurements, it is also possible to calculate the maximum TE power output of the TEG GFRP laminate according to the following Equation (5) [28]:

(5) $$P_{max}=\frac{V_{TEG}{}^{2}}{4xR_{TEG}}$$

where:

*P_max_* = maximum power output in nW
*V_TEG_* = the TE open-circuit voltage in mV
*R_TEG_* = internal electrical resistance of the TEG in Ohm

Oriented to a TEG device characterization for practical applications, power output measurements as a function of the externally applied load resistances (*R_LOAD_*) were carried out. Thus, the through-thickness TEG GFRP device power output characteristics have been evaluated using a custom-built fully automated electronic system based on a LabVIEW-PC interface with a range of applied external loads from 1 to 10,000 Ohm with discretion capability down to 1 Ohm. Apart from the experimental power output values, it is possible to obtain calculated ones using Equation (6) [52]:

(6) $$P=I^{2}xR_{LOAD}={\left(\frac{V_{TEG}}{R_{TEG}+R_{LOAD}}\right)}^{2}xR_{LOAD}$$

where:

*P* = the output power in nW
*I* = the output current that passes through the load in μA
*R_Load_* = externally applied load resistances in Ohm

All tests were performed at ambient conditions (1 atm, T_C_ ~ 25 °C, relative humidity: 40 ± 5% RH).

Finally, the mechanical performance of the unmodified and TEG GFRP laminates was evaluated under flexural loading according to ASTM D 790-03 standard [53] using a 100 KN Universal Testing Machine (Jinan Testing Equipment IE Corporation, Jinan, China). Five rectangular specimens (50 × 10 × ~2.6 mm^3^) were tested for each type of laminate at a deformation rate of 1 mm/min. All specimens were conditioned in an oven at 40 °C overnight prior to testing.

## 3. Results and Discussion

### 3.1. Characterization of the Inorganic-Organic Nanomaterial and the Coated GF Fabric

**Figure 4:**
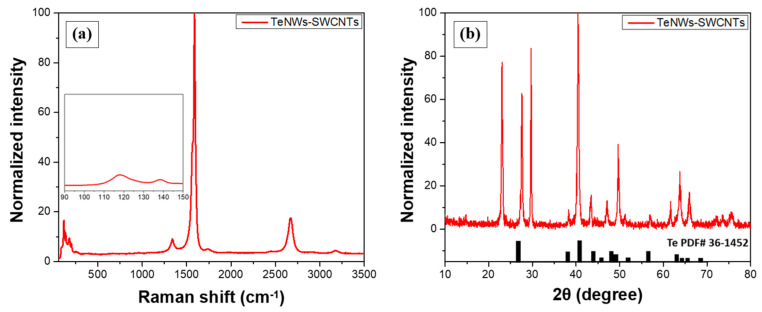
(**a**) Raman spectrum and (**b**) X-ray diffraction pattern of the TeNWs-SWCNTs buckypaper film.

Raman and XRD spectra were obtained from the developed buckypaper films to verify the presence of hybrid TeNWs-SWCNTs inorganic-organic thermoelectric material. As illustrated in Figure 4a, the existence of TeNWs is identified through the Raman peaks at 118.1 cm^−1^ and 138.2 cm^−1^. Furthermore, the peak at ca. 120 cm^−1^ is attributed to the Te content and the A_1_ vibrational mode response of TeNWs [54]. SWCNTs are also visible via the D (1337 cm^−1^), G (1588 cm^−1^), and 2D (2666 cm^−1^) peaks. A characteristic peak at ca. 1588 cm^−1^ is related to the vibration of sp^2^-bonded and in-plane stretching E_2g_ mode of carbon atoms of the SWCNTs. Raman spectra of SWCNTs located this characteristic peat at ca. 1590 cm^−1^ [30]. As discussed previously, the slight shift of the peak position of the G-band can be attributed to the interaction between the TeNWs with SWCNTs, leading to a decreased conjugation [47]. A relatively lower peak at ca. 1337 cm^−1^ is correlated with sp^3^ hybridization [55].

Moreover, as illustrated in Figure 4b, the XRD pattern of the inorganic-organic buckypaper film is in agreement with the Te reference spectrum (36–1452, black inset bars) [49]. Thus, the XRD spectra confirmed the crystal hexagonal structure of tellurium with three atoms per unit cell and cell constants equal to 4.46 Å for a and 5.92 Å for c [56].

For comparison purposes, the in-plane TE properties of the reference inorganic and the developed inorganic-organic buckypaper film are included in Table 1. The reported mean TE values are referred to five measurements of different buckypaper films for each case. The positive values of the Seebeck coefficient indicate the p-type semiconducting behavior for both materials. Furthermore, as observed in Table 1, the TeNWs-based film presented a relatively high Seebeck coefficient of approximately +302 μV/K combined with a low electrical conductivity of 8.4 S/m, which corresponds to a *PF* of 0.77 μW/m·K^2^. At the same time, the inorganic-organic film presented a Seebeck coefficient of approximately +80 μV/K combined with an electrical conductivity of 9200 S/m, resulting in a two order of magnitude higher *PF* of 58.88 μW/m·K^2^, compared to the inorganic TE film. Since κ values were not experimentally, the calculation of the *ZT* values was obtained using respective values from other studies [48] (see Table 1). We believe that this is a good approximation since similar synthetic routes were followed for the manufacturing of the TeNWs and TeNWs-SWCNTs as those presented in [48]. It should also be noted that an average value of *ZT* was obtained since the actual temperature was approximated with the externally applied Δ*T*. As observed, the energy conversion efficiency for the reference system is as low as 0.008%. On the contrary, the combination of the TeNWs with SWCNTs resulted in approximately 74 times higher efficiency of the obtained TE nanomaterials. Based on the above, it can be concluded that the addition of the highly conductive SWCNTs within the TeNWs was beneficial for the overall TE performance of the developed films. This can be attributed to bridging phenomena that create conductive paths between the two nanomaterials, resulting in a reduction of the contact resistance without eliminating the overall TE performance [57,58,59]. Moreover, the extremely brittle nature of TeNWs buckypaper films renders this material unsuitable for further processing and practical applications. Therefore, the inorganic-organic nanomaterial was employed for TE ink production.

**Figure 5:**
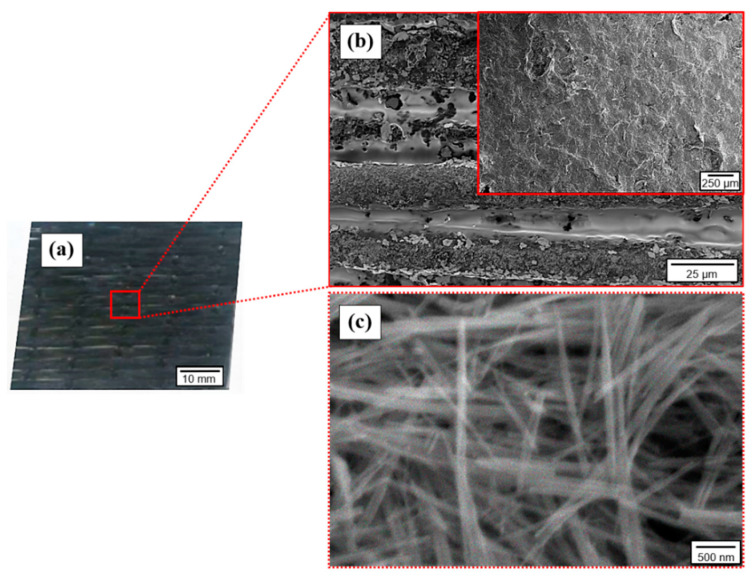
(**a**) Image of the TE-coated GF and (**b**,**c**) SEM images at different magnifications of the coated GF ply.

Images of the TE-coated GF fabric at different magnifications are presented in Figure 5. Based on these images, satisfying adhesion properties between the TE coating and the fibrous substrate is elucidated by the continuously distributed nanostructures onto the surface of the GFs. Consequently, the created through-thickness uniform coating introduces multiple interconnected TE paths due to GF fabric porosity and voids between the stitched GF tows, as observed in Figure 5a,b. Subsequently, the highest magnification image (Figure 5c) reveals a dense network of high aspect ratio typical 1D nanostructures with a diameter of a few nm, which correspond to the TeNWs-SWCNTs hybrid system. The absence of any aggregates implies the preparation of a high-quality TE ink and the application of an efficient coating process. This is expected to result in superior bulk TE properties of the coated GF fabric.

### 3.2. Characterization of the TEG GFRP Laminate

Table 2 presents the experimentally measured TE values and the calculated maximum TE power output of the TEG GFRP laminate at different thermal gradients. The average output values correspond to TE measurements, which arose from four manufactured TEG GFRP laminates. At this point, it is worth mentioning that the measured through-thickness internal electrical resistance (*R_TEG_*) value of the laminate system prior to the impregnation of the epoxy resin was as low as ~3 Ohm at R*T*. Subsequently, after the hardening of the epoxy matrix, the composite laminates’ *R_TEG_* value was slightly increased at 8.3 Ohm. This increase could be mainly ascribed to possible interactions between the epoxy resin, the thin coating of the TE GF lamina, and the Ag-coated GF laminae. Thus, the internal TEG interconnection marginally affected the electrical characteristics of the manufactured laminates.

As observed in Table 2, the manufactured through-thickness TEG GFRP laminate can harvest thermal energy upon exposure to a temperature gradient and under enforced cooling to sustain the design desired Δ*T*. In the case where there is no enforced cooling, the power output of the TEG appears to be insufficient. For instance, a Δ*T* of 50 K generates a sufficient open-circuit voltage (Seebeck voltage) of 0.76 mV, which increases with a further increase of the Δ*T*. Inevitably, a short-circuit current of 82.67 μA and a power output of 15.78 nW could be achieved at a temperature difference of 50 K that can be easily attained between a car engine in operation and the outside air that surrounds the car during its movement [60,61]. The maximum power output of 54.22 nW of the through-thickness TEG GFRP laminate, derived at Δ*T* = 100 K, corresponds to a power density of 0.02 W/m^2^. The power density value was calculated by dividing the maximum TE power output with the cross-sectional active area of the coated GF ply.

**Figure 6:**
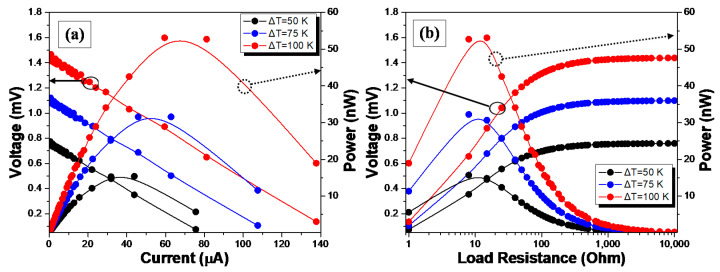
(**a**,**b**) Power output characteristics for Δ*T* of 50, 75, and 100 K.

Figure 6a,b, shows the power output characteristics for the TEG GFRP laminate. In more detail, the multifunctional GFRP laminate exhibits an open-circuit voltage (*V_TΕG_*) of 1.44 mV and short-circuit current (I_SC_) of 154.6 μA at Δ*T* of 100 K with an internal resistance (*R_TEG_*) of 9.56 Ohm. Figure 6a depicts the measured TE performance in various Δ*T.* Specifically, output voltage-current (V-I), output power-current (P-I) curves. Figure 6b depicts the output voltage-external load (V-*R_LOAD_*) and output power-external load (P-*R_LOAD_*) curves with the application of different external load resistances. The continuous lines in all cases have been derived from calculations, while the dots correspond to the experimental values that were acquired through the specially designed custom-built measuring unit. A maximum power generation of 54.22 nW at a through-thickness Δ*T* of 100 K is dissipated at the applied external electrically in-series connected *R_LOAD_*. As it was noticed, when the external load is compatible with the *R_TEG_* experimental value of 9.53 Ohm, the maximum output power is matched with the external load resistance of ca. 9.5 Ohm, which is equal to the *R_TEG_* value. As expected, the output voltage for the different *R_LOAD_* was inversely proportional to the output current, presenting a typical parabolic behavior.

Conventional bulk or micro-scale through-thickness TEG designs during application suffer from thermal gradient equilibrium during time evolution, especially in the case where the heat dissipation is spontaneous, without being sustained artificially [21,59]. For comparison purposes, it is important to mention that the in-plane TE output of the inorganic-organic buckypaper was 8 ± 0.12 mV, at 25 mm for an applied Δ*T* of 100 K. The respective through-thickness TE voltage of the TEG laminate was 1.44 mV, as stated in Table 2. Thus, the TEG laminate shows a lower through-thickness voltage by ~82% compared to the voltage obtained from the in-plane measurement of the buckypaper. This could be attributed mainly to the extremely compact interelectrode distance ~0.27 mm in the case of the TEG laminate, as has also been previously pointed out, e.g., [41]. Partially, the thermal insulating character of the GFRP laminate could also negatively affect the internal Δ*T* distribution, resulting in lower power output values in relation to the expected ones. Additionally, it is worth noting that the abovementioned values are the result of a single thermoelement. Indicative, Inayat et al. succeeded a TE power output of 112 nW for a Δ*T* οf ~20 K resulting from a four-thermoelement inorganic nanomaterial-based through-thickness TEG prototype window glass [62]. Similarly, Lu et al. developed a fabric-based through-thickness TEG prototype consisting of 12 inorganic nanostructured thermoelements that were able to achieve a TE power output of ca. 15 nW at a Δ*T* οf ~30 K [63].

Based on the above, it is obvious that the developed laminate has the potential for significant thermal energy harvesting. This can be further optimized after the fabrication of in-series or in-parallel interconnected modules of thermoelements to increase the total TE power generation. It is therefore demonstrated that the exploitation of through-thickness TEG GFRP composite laminates could realize effective thermal energy harvesting power by structural components.

**Figure 7:**
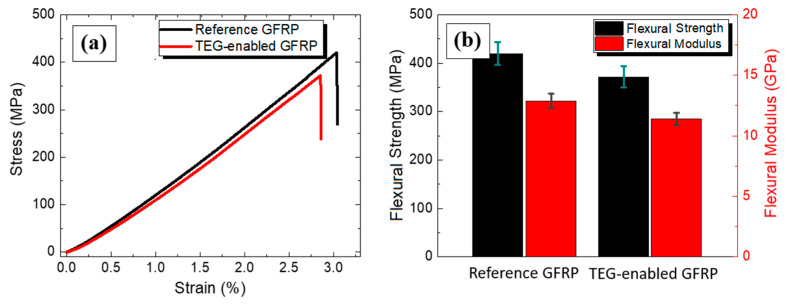
Comparison plots for the reference GFRP and the through-thickness TEG GFRP specimens (**a**) representative stress-strain curves and (**b**) average flexural strength and modulus.

The mechanical performance of the multifunctional GFRP laminate was evaluated under flexural loading and compared with the plain reference laminate. Based on representative stress-strain curves of the tested specimens, it can be observed that the functional laminate behaves similarly with the reference GFRP, as shown in Figure 7a. The stress-strain curve of the TEG laminate lies slightly below one of the reference materials. The stress-strain curves of all tested specimens were assessed to calculate the average flexural modulus and strength before and after the GFRP modification. Based on the results presented in Figure 7b, the flexural strength was 371.70 ± 23.43 MPa, and the flexural modulus was 11.41 ± 0.5 GPa for the multifunctional GFRP, while the respective values for the plain GFRP were 420.04 ± 24.47 MPa and 12.89 ± 0.6 GPa. Thus, it can be concluded that the integration of the functional GF ply and the Ag-coated plies resulted in a minor alteration of the response of the functional laminate and a respective reduction of ~11.5% in both the strength and the modulus.

The obtained mechanical results disclose relatively equivalent structures according to the flexural strength and the modulus. The requirement of both metallic and TE functional coatings onto specific GF plies contributes to the slightly decreased mechanical properties; however, the laminates maintained to a great extent their advanced properties and can still be used as structural composites.

## 4. Conclusions

The scope of this research was to introduce through-thickness thermal energy harvesting capabilities to conventional GFRP composite laminates without eliminating their structural integrity. For this reason, an efficient, flexible p-type inorganic-organic TE nanomaterial was synthesized and further processed to produce aqueous TE ink for dip-coating purposes creating hierarchically coated GF UD reinforcement fabrics. The resulting coated functional GF ply was employed for the first time to manufacture a through-thickness TEG-enabled GFRP laminate, which exhibited a power output of 54.22 nW from a single thermoelement upon exposure to a through-thickness Δ*T* of 100 K. Regarding the mechanical performance, the multifunctional structure displayed slightly decreased values ca. 11.5% of bending strength and flexural modulus with respect to the reference GFRP laminates.

Future developments in flexible, chemically stable, and environmental-friendly TE materials with enhanced *ZT* values in the range of ~1 at reasonable temperature gradients could dramatically improve the power output characteristics at the material level, oriented to prospect practical applications. Thus, the fabrication at the device level of multiple in-series and/or in-parallel interconnected modules of thermoelements could further stimulate the total generated TE power from composite structures. Thermal energy harvesting and conversion by structural materials with promising power output in the range of several microwatts is sufficient to power up external step-up low power-consuming converters for the energy storage, leading to exploitable energy management and use toward the activation of low-power electronics such as wireless sensor networks, etc. Based on the above, power generation by structural engineering materials designed to be routinely exposed to temperature gradient could emerge as an attractive technology for the realization of large-scale thermal energy harvesting applications in various industrial sectors.

## Figures and Tables

**Table 1:** TE values of the inorganic and inorganic-organic buckypaper films at Δ*T* of 100 K. Note that κ values used to calculate ZT are taken from elsewhere [48].

TE Material	*σ*	*S*	*PF*	κ	*ZT*	η
	S/m	μV/K	μW/m·K^2^	W/(m·K)	-	%
TeNWs	8.4 ± 0.6	+302 ± 8	0.77	0.28	0.001	0.008
TeNWs-SWCNTs	9200 ± 5	+80 ± 4	58.88	0.26	0.080	0.590

**Table 2:** TE measurements of the through-thickness TEG GFRP laminate at various Δ*T*.

Δ*T* (*K*)	*R_TEG_* (Ohm)	*V_TEG_* (mV)	I_sc_ (μA)	*P_max_* (nW)
0	8.30 ± 0.10	-	-	-
50	9.15 ± 0.15	0.76 ± 0.13	82.67 ± 0.18	15.78
75	9.33 ± 0.24	1.10 ± 0.17	119.84 ± 0.24	32.42
100	9.56 ± 0.32	1.44 ± 0.22	154.60 ± 0.38	54.22

## Data Availability

The data presented in this study are available on request from the corresponding author. The data are not publicly available due to privacy.
